# Musculoskeletal disorders in Norway: trends in health care utilization and patient pathways: a nationwide register study

**DOI:** 10.1080/02813432.2024.2368848

**Published:** 2024-07-21

**Authors:** Mari Kristine Tyrdal, Flavie Perrier, Cecilie Røe, Bård Natvig, Astrid Klopstad Wahl, Marit B. Veierød, Hilde Stendal Robinson

**Affiliations:** a Department of Interdisciplinary Health Sciences, Institute of Health and Society, University of Oslo, Oslo, Norway; b Oslo Centre for Biostatistics and Epidemiology, Department of Biostatistics, Institute of Basic Medical Sciences, University of Oslo, Oslo, Norway; c Department of Physical Medicine and Rehabilitation, Oslo University Hospital, Oslo, Norway; d Department of General Practice, Institute of Health and Society, University of Oslo, Oslo, Norway

**Keywords:** Register data, primary health care, general practitioner, physiotherapy, specialist health care

## Abstract

**Objective:**

Describe trends in health care utilization, demographic characteristics and patient pathways among patients with musculoskeletal disorders (MSD) in Norway.

**Design:**

Register-based cohort study.

**Settings:**

Data were obtained from two Norwegian National registries; the Norwegian Control and Payment of Health Reimbursements Database (KUHR) and the Norwegian Patient Registry (NPR).

**Subjects:**

Patients with MSD according to ICPC-2 and ICD-10 during 2014–2017.

**Main outcome measures:**

Patient pathways from the first contact and the following two years, described in a Sankey Diagram for all MSD patients and three common diagnoses: spine pain, osteoarthritis (OA) and fibromyalgia (FM).

**Result:**

About 26% of the Norwegian population consulted PHC annually while 7% were treated in SHC. Mean age was 47 and 53 years in PHC and SHC, respectively. The proportion of women increased by age. Spine pain was the most common diagnosis; 33% and 22% in PHC and SHC, respectively. Over 90% visited a GP first, 50% of them were treated by PT and/or in SHC during follow-up. Patients visiting the PT first were less likely to be treated in SHC. OA patients were most likely to be treated by more than one health care professional (>70%).

**Conclusion:**

One third of the Norwegian population consulted health care services due to MSD annually between 2014–2017. GP was the most consulted health care professional. Among MSD patients with long-term use of health care services, 50% were treated by a PT and/or in SHC in addition to a GP.

## Introduction

Musculoskeletal disorder (MSD) is the leading cause of years lost to disability with a significant burden on both individuals and societies [1, 2]. In the European Union (EU), MSD is the most common work-related health problem, causing the highest productivity loss, compared to all other non-fatal conditions [3, 4]. First-line treatment of MSD is commonly provided in primary health care (PHC), mainly by general practitioners (GPs) [5, 6]. Persistent pain may lead to referral to other health care professionals, e.g. physiotherapists (PT) or medical/physician specialists [7–9]. Hence, high prevalence also causes considerable health care costs. Moreover, a small percentage of the patients counts for most of the expenses [10, 11], with inpatient services in specialist health care (SHC) as most costly [6].

In Norway, the prevalence of MSD is among the highest in the world [12, 13] with substantial impact on health care services. About one third of all PHC contacts are caused by MSD [14]. With a growing and ageing population, the burden of MSD will hardly decrease in the years to come, and with the predicted shortage of health care workers in the future, prioritization of health care services will be enforced. The knowledge about health care utilization and patient pathways among MSD patients is scarce. However, Norway with nationwide registers for provided services has unique sources to such knowledge.

All Norwegian inhabitants (5.5 million) are provided with public services irrespective of a private insurance or not. They are entitled to have a GP, which offers extensive care for a broad range of health issues and is a gatekeeper for more specialized care. Until 2018, a referral was required to receive treatment from PTs, except for manual therapists (MT; PTs specialized in musculoskeletal conditions). Patients receive free health care except for an annual maximum payment of about 240 USD (2018). The government keeps track of all health care use with several mandatory national health registers. The unique personal identification number of every Norwegian citizen enables linking data from different registers and to track patients over time. The registered diagnosis codes enable distinguishing between diagnoses. This gives a unique possibility to describe healthcare care utilization and patient pathways for MSDs. The treatment strategies for MSD often differ and the lack of guideline implementation may lead to both random use of health care and referral practice [15, 16]. Studies have called for improvement in the quality of care in PHC of MSDs [17, 18] and a Lancet report addressed the gap between evidence and practice leading to unnecessary use of health care services [19]. For this study, we wanted to explore possible differences in health care utilization and patient pathways for MSD in general, and for the following three common diagnoses easily identified in the national registers: spine pain, osteoarthritis (OA) and fibromyalgia (FM).

Our aim was to use Norwegian nationwide register data to (1) describe trends in health care utilization and demographic characteristics of MSD patients diagnosed from 2014 throughout 2017, and (2) describe patient pathways over a two-year period for all MSD patients and for the three diagnosis groups, spine pain, OA and FM.

## Materials and method

### Data sources

This is a register-based cohort study with data from two national registers in the period 2014 throughout 2017. To answer the first aim, we counted annual number of unique patients and their use of health care services each year. For the second aim, we followed patients treated for MSD in PHC in 2015 (without consultations in 2014) for 2 years.

### The Norwegian control and payment of health reimbursements database (KUHR)

KUHR is a registry covering all health care contacts provided in the publicly funded PHC. Services provided in PHC are organized by the municipalities in Norway. KUHR contains data about reimbursements from contacts by GPs, PTs (including MTs), chiropractors and in emergency outpatient clinics. In this study, we used only information from the GPs and the PTs. Every consultation is assigned with at least one diagnosis according to the 2^nd^ edition of the International Classification of Primary Care (ICPC-2).

### The Norwegian Patient Registry (NPR)

The NPR is a national registry, covering all patient-contacts in publicly funded hospitals and medical specialists outside hospitals. In Norway, four regional health authorities provide specialist health care services in their respective regions. In NPR, contacts are assigned to be either outpatient, inpatient or day patients with minimum one diagnostic code according to the International Classification of Diseases 10^th^ edition (IDC-10).

### Ethics

This project was approved by the Regional Committee for Medical and Health Research Ethics in Norway (2018-1280-1).

### Study population

**Figure 1.**
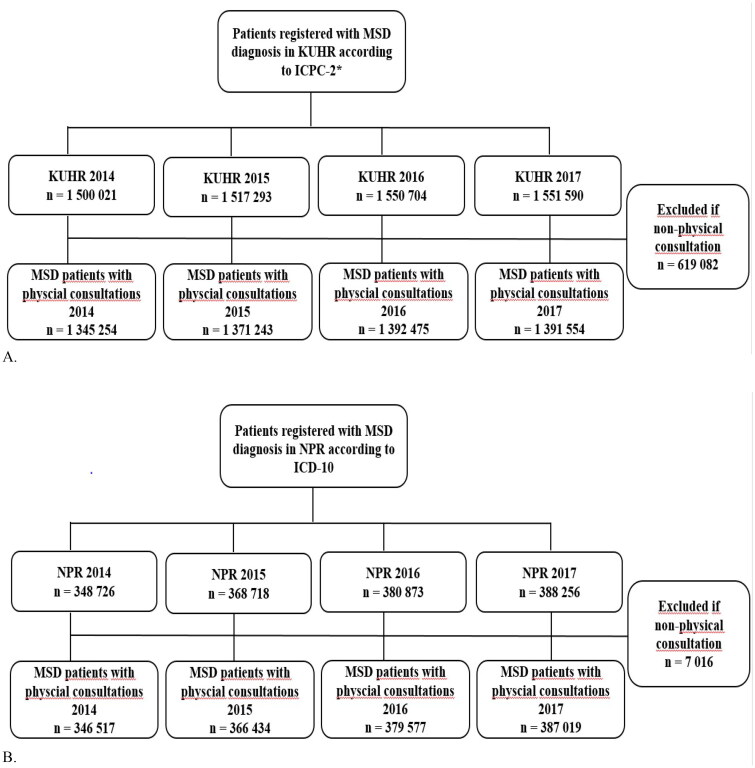
Flow diagram for inclusion of patients with musculoskeletal disorders (MSD) registered between 2014 and 2017 in (A) the Norwegian control and payment of health reimbursements database (KUHR; primary health care) and (B) the Norwegian Patient Registry (NPR; specialist health care). *Consultations only by general practitioner (GP) or physiotherapist (PT).

To assess the extent of health care utilization, we identified all individuals with GP and/or PT contacts with musculoskeletal disorders, L-codes in KUHR, and accordingly for SHC all contacts with M-codes in NPR between 2014 and 2017 (Figure 1). Consultations registered as non-physical consultations (e.g. telephone or e-mail consultations) according to the reimbursement rate were excluded. After this exclusion, it was between 1,345,000–1,390,000 patients with L-codes in KUHR and 346,000–387,000 patients with M-codes in NPR registered each year. These were included in the study population for the analysis of health care utilization and demographic characteristics.

**Figure 2.**
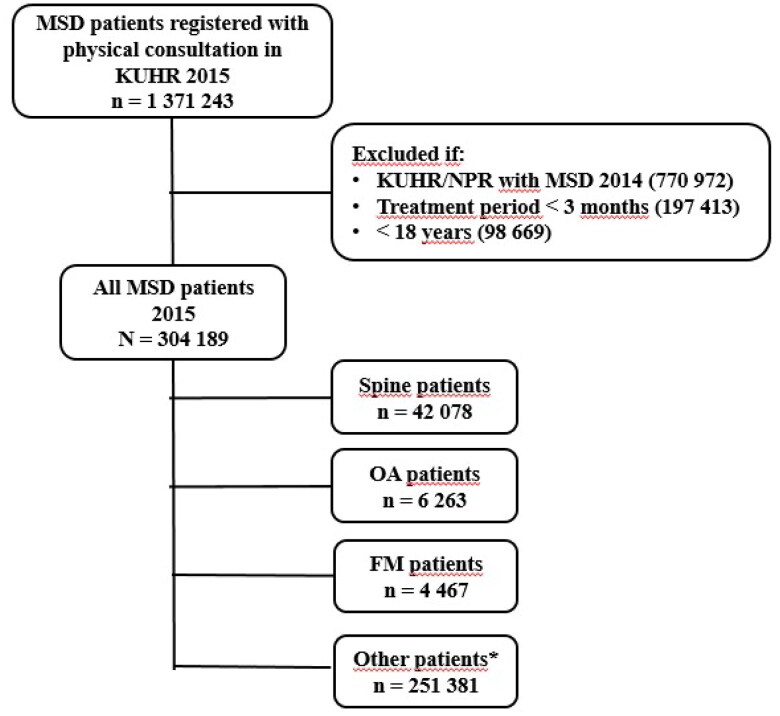
Flow diagram for inclusion of musculoskeletal disorders (MSD) patients to the analysis of patient pathways from the Norwegian control and payment of health reimbursements database (KUHR; primary health care) 2015. *Not included in the analysis of patient pathways due to inclusion criteria

To analyze patient pathways, we established a cohort of MSD patients identified by the L-codes in KUHR in 2015 (Figure 2) and linked them to data from the NPR. The first contact should be in PHC, either by GP or PT. Due to the referral system, a first contact registered with the PT is actual a MT. Baseline was the first date of consultation in 2015 with either a GP or PT, and time from baseline to the next consultations (GP, PT and SHC) within the following two years (total 730 days) was calculated. Patients registered with consultations due to MSD during the 12 months prior to baseline, were excluded from the analyses. In addition, patients should have two or more consultations over at least a 3-month period and be aged ≥18 years to be included (Figure 2). In total, 304,189 MSD patients were included in the analyses of patient pathways, 42,078 patients with spine pain (L01-L03/L83-L86 and M40-54), 6263 patients with OA (L89-91 and M15-19) and 4467 patients with FM (L18 and M79) (Figure 2). Patients should have the relevant diagnosis registered at both the first and last consultation in PHC.

### Variables

For each consultation we extracted information about age, sex (woman, man), health care profession visited (GP and PT in KUHR and SHC in NPR), date, reimbursement rate and diagnosis code for each consultation from KUHR and NPR. Consultations in SHC were registered as either outpatient, inpatient or day patients. To receive payment, at least one diagnosis code must be registered. Even though patients may present several complaints and comorbidities, usually only one diagnosis is reported.

When we linked KUHR and NPR for the analysis of the patient pathways, age and sex at baseline from KUHR was used. Patient characteristics for all MSD patients and the three diagnosis groups (spine, OA and FM), are presented according to the first consultation being with a GP or PT.

### Statistical analyses

For KUHR and NPR, patients characteristics by year of consultation from 2014 to 2017 are presented as frequencies and percentages (%), means with standard deviations (SDs) or median and interquartile range (IQR, 25% −75%) as appropriate.

Sankey diagrams (SankeyMATIC) were used to describe the patient pathways. The columns represent the proportion of patients visiting different health care professionals; GP and PT in the first column and GP, PT and SHC in the second and third column. Patients could be included in only one pathway, based on first consultation with either GP or PT, giving ten possible pathways.

The data were analyzed using STATA (version 17, StataCorp. Texas, USA).

## Results

### Healthcare utilization

**Figure 3.**
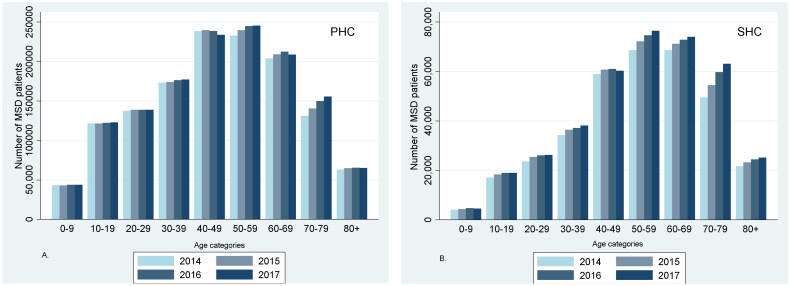
The total number of patients in (A) the Norwegian control and payment of health reimbursements database (KUHR; primary health care (PHC)) and (B) the Norwegian Patient Registry (NPR; specialist health care (SHC)) each year from 2014 to 2017, divided by age category.

Annually from 2014 to 2017, approximately 26% of the Norwegian population visited health care professionals in PHC due to MSD. In the same period, 7% of the Norwegian population was treated annually in SHC for MSD (Table 1). Mean (SD) age for MSD patients treated in PHC and SHC was consistent in the study period (Table 1). Regarding age, 40–49 and 50–59 years were the most frequent age decades for patients treated in PHC and 50–59 and 60–69 years in SHC (Figure 3). During the study period, the annual number of patients in the age decade 70–79 years increased, both in PHC and SHC.

**Table 1.** Characteristics for patients with musculoskeletal diagnoses (MSD) registered in the Norwegian control and Payment of health reimbursements database (KUHR; primary health care) consulting general practitioners (GP) and/or physiotherapist (PT) only, and in the Norwegian Patient Registry (NPR; specialist health care (SHC)**)**, from 2014 throughout 2017.

	2014		2015		2016		2017	
Health register	KUHR	NPR	KUHR	NPR	KUHR	NPR	KUHR	NPR
Number of patients	1 345 254	346 517	1 371 243	366 434	1 392 475	379 577	1 391 554	387 019
Age, mean (SD)*	46.8 (20.7)	53.1 (19.0)	47.2 (20.7)	53.2 (19.2)	47.4 (20.7)	53.5 (19.3)	47.4 (20.8)	53.7 (19.2)
Women, n (%)	758 144 (56.4)	205 401 (59.3)	770 690 (56.0)	217 256 (59.3)	783 651 (56.3)	224 314 (59.1)	782 722 (56.3)	229 026 (59.2)
Diagnoses, n (%)								
Spine pain	448 409 (33.3)	74 270 (21.4)	474 573 (34.6)	80 067 (21.9)	458 422 (32.9)	82 251 (21.7)	457 528 (32.9)	82 824 (21.4)
Osteoarthritis	139 040 (10.3)	68 306 (19.7)	145 550 (10.6)	72 075 (19.7)	151 501 (10.9)	75 167 (19.8)	138 509 (10.0)	75 955 (19.6)
Fibromyalgia	97 082 (7.2)	44 507 (12.8)	100 707 (7.3)	47 912 (13.1)	102 674 (7.4)	50 123 (13.2)	103 193 (7.4)	51 742 (13.4)
*Norwegian population*	*5 109 056*		*5 165 802*		*5 213 985*		*5 258 317*	

* SD: standard deviation.

**Figure 4.**
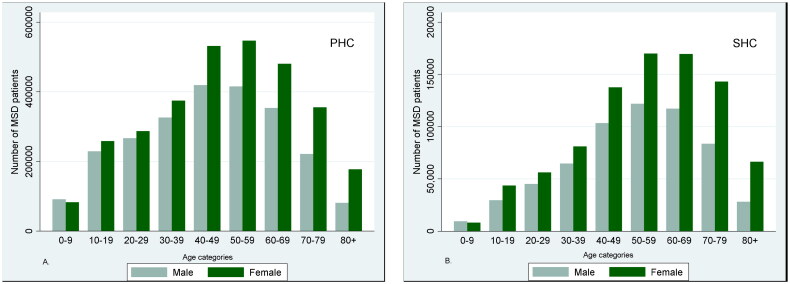
The total number of patients registered in (A) the Norwegian control and payment of health reimbursements database (KUHR; primary health care (PHC)) and (B) the Norwegian Patient Registry (NPR; specialist health care (SHC)) between 2014 and 2017 with musculoskeletal diagnoses (MSD), divided by age category and gender.

A higher proportion of women visited health care services due to MSD compared to men (Table 1), and the difference between sexes increased with age (Figure 4). About 33% and 22% of the patients were registered with a spine diagnosis in PHC and SHC, respectively (Table 1). The corresponding proportions for OA were 11% and 20% and for FM 7% and 13%.

**Figure 5.**
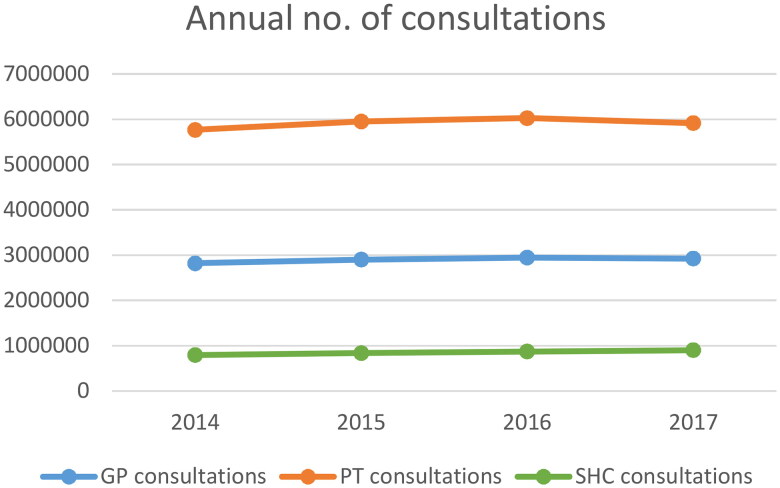
Annual number of physical consultations by general practitioner (GP) and physiotherapist (PT) registered in the Norwegian control and Payment of health reimbursements database (KUHR; primary health care) and specialist health care (SHC) registered in the Norwegian Patient Registry (NPR).

Among patients with at least one GP consultation, the median (IQR) annual number of GP consultations per patients was 1 (1–3). The median (IQR) annual number of PT consultations for those with at least one PT consultation was 9 (4–22) and the corresponding annual number in SHC was 1(1–2). Annually, about 3 and 6 million consultations with GPs and PTs respectively were due to MSD (Figure 5). In SHC the annual number of consultations was approximately 900 000. Outpatient consultations represented more than 80%, while inpatient and day patient consultations were less common (7 and 13%, respectively).

### Patient pathways

Mean age was almost the same for MSD patients consulting a GP or PT first (49 and 50 years, respectively) (Table 2). Among the diagnosis groups, OA patients were the oldest (67 and 68 years, for GP and PT first, respectively) and spine patients were the youngest (46 years for both). FM patients had the highest proportion of women (GP first: 78%; PT first: 82%). Furthermore, a higher proportion of women consulted a PT first in all the three diagnosis groups, and the largest sex difference was found among spine patients (GP first: 51% women; PT first: 62% women) (Table 2).

**Table 2.** Patients characteristics, number of consultations and time from general practitioner (GP)/physiotherapist (PT) to GP/PT/specialist health care (SHC) for all patients with musculoskeletal diagnoses (MSD) diagnosed in 2015 and for the three selected diagnosis groups, by first consultation in primary health care.

	All MSD diagnosis		Spine pain		Osteoarthritis		Fibromyalgia	
	304 189		42 078		6 263		4 467	
Number of patients	GP first	PT first	GP first	PT first	GP first	PT first	GP first	PT first
Number of patients	285 277	18 913	37 722	4 356	5 460	772	4 142	325
Age, mean (SD)	49.3 (17.4)	50.1 (17.7)	45.7 (16.4)	46.0 (16.5)	67.4 (12.1)	67.7 (11.7)	47.0 (14.8)	47.7 (15.7)
Women, n (%)	151 588 (53.1)	11 105 (58.7)	19 329 (51.2)	2 688 (61.7)	3 293 (60.3)	474 (61.4)	3 219 (77.7)	267 (82.2)
Total cons*, median (IQR)**	5 (3–10)	13 (7–27)	5 (3–11)	12 (6–24)	7 (3–25)	35 (18–63)	5 (3–11)	20 (11–38)
GP cons, median (IQR)	3 (2–5)	1 (0–3)	3 (2–6)	1(0–2)	3 (2–5)	2 (0–3)	3 (2–6)	0 (0–2)
PT cons, median (IQR)	0 (0–2)	11 (5–24)	0 (0–3)	10 (5–22)	0 (0–17)	32 (15–59)	0 (0–1)	18 (10–36)
SHC cons, median (IQR)	0 (0–1)	0 (0–0)	0 (0–1)	0 (0–0)	1 (0–3)	0 (0–1)	0 (0–1)	0 (0–0)
Number of days from first GP to first PT, median (IQR)	174 (56–405)		135 (40–361)		150 (53–330)		127 (34–316)	
Number of days from first GP to first SHC, median (IQR)	204 (98–417)		207 (104–408)		146 (71–349)		245 (110–448)	
Number of days from first PT to first GP, median (IQR)		215 (75–424)		224 (87–427)		167 (71–356)		185 (51–391)
Number of days from first PT to first SHC, median (IQR)		279 (130–476)		307 (161–501)		238 (123–435)		338 (151–533)

* Consultations.

** IQR: interquartile range, 25–75 percentile.

Median (IQR) number of consultations for all MSD patients was 5(3–10) for those visiting the GP first and 13(7–27) for the PT first. Patients visiting a PT first had fewer GP consultations than those visiting a GP first (median (IQR), 1(0–3) versus 3(2–5)). OA patients had more consultations in total compared to spine and FM patients (median (IQR), 7(3–25) for GP first and 35(18–63) for PT first). The median number of GP consultations was similar in the three diagnosis groups (Table 2).

FM patients had shorter time (median (IQR)) between the first GP consultation and the first PT consultation (127(34–316) days) compared to spine and OA patients. OA patients had the shortest time from the first GP visit to the first SHC consultation (146(71–349) days). This was almost similar to the time from the first GP consultation to the first PT consultation (150(53–330)). For all patients, the time between the first visit to a GP and to other health care professionals was shorter than for those consulting a PT first.

**Figure 6.**
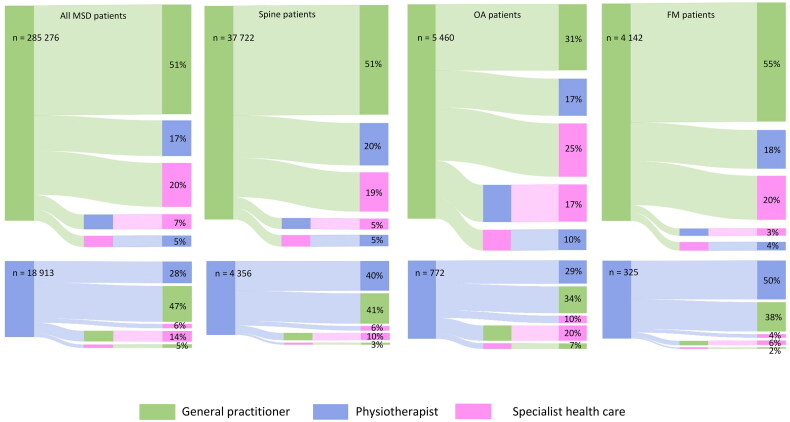
Sankey diagram describing patient pathways for all musculoskeletal disorders (MSD) patients as well as the diagnosis groups: spine, osteoarthritis (OA) and fibromyalgia (FM), from baseline date in 2015 and two years ahead.

A total of 94% of the patients consulted a GP first (Figure 6). Among these, 51% visited no other health care professionals during the two-year follow-up, 17% and 20% had at least one additional consultation (with a PT or in the SHC respectively), while 12% visited both a PT and the SHC. Among the 6% (18,913) of MSD patients visiting a PT first, 28% visited no other health care professionals, 47% and 6% consulted a GP or the SHC, respectively, while 19% consulted both a PT and the SHC. A lower number of patients were treated in SHC after visiting a PT first (25%) compared to those visiting a GP first (32%).

No differences were found in the proportion of patients visiting a GP or PT first in the three diagnosis groups. The number of patients visiting more than one health care professional was highest in the OA group and lowest in the FM group. OA patients were most frequently treated in the SHC. The treatment rate in SHC was lower after visiting the PT first compared to GP first in all the diagnosis groups (Figure 6).

## Discussion

We found that about one third of the Norwegian population visited a health care professional annually from 2014–2017 due to MSD. Among the selected diagnosis groups, spine pain was the most common. Moreover, the use of health care services in publicly funded PHC and SHC were consistent during the study period. Most of the patients were in the age groups between 40–60 years, and the proportion of women increased by age. More than 90% of the MSD patient visited a GP first and half of them did not seek any other health care professional during the next two years. OA patients were most likely to be treated by a PT and/or in SHC after visiting the GP while spine and FM patients were more likely to visit only the GP.

### Strengths and limitations

A major strength of this study is the use of nationwide complete health registers. These registers are representative for the Norwegian population with no loss to follow-up. The unique identification number of all citizens in Norway allows linkage of data from different registers and thereby makes it possible to follow patients in different levels of health care services. Due to a long application process, the data are from 2014–2017. However, we consider the MSD patients to be representative and the patient pathways to be of interest despite the changes in the regulations regards to referral. The use of these registers excludes the risk of recall/self-reported bias. However, we lack information about patients using private clinics in PHC and SHC. Although the Norwegian health care system is mostly publicly funded with the GP as gatekeepers, private insurances and offerors are growing and consultations in private clinics will not appear in the registers. Moreover, information about treatment of elderly persons in the nursing homes is not included in the KUHR database, and this may give some explanation to the lower number of elderlies with MSD in PHC (Figure 3).

Using a “washout” period of at least one year as a definition of “new” incidence of MSD and the follow-up period of two years in the analysis of patient pathways might have been short. The diagnoses are based on ICPC and ICD-codes. These codes are registered mainly for administrative claims (payment). Generally, these claims include only one diagnosis disregarding that the patient might present with several diseases/diagnoses. Moreover, registration error may also occur. Estimating actual prevalence of diseases can therefore be somewhat misleading. However, these codes have often been used as reliable classification systems and for comparison of groups in primary and specialist health care [20]. Lastly, since we have analyzed register data, we have no information about the content of the treatment given by any of the health care providers.

### Health care utilization

Our results support previous studies in that MSD is a major burden on the health care system [21, 22]. The proportion of patients seeking health care annually due to MSD in our study, is also in accordance with a previous register-based study from Norway [14]. Both studies found that the proportion of MSD patients in the publicly funded health care system was consistent over the years studied. However, as up to 40% of persons with MSD never seek health care services [11] and the use of private health care services increases in Norway [7], the prevalence of MSD is probably higher. The total annual number of consultations was also consistent over the study period and PT consultations were the largest contributor. This was not surprising, since treatment by PT usually involve more than one consultation and most often indicate follow-up over some time. Moreover, studies have also found that follow-up by PT gave better outcome and was cheaper due to less imaging, less medial prescription and referrals compared to follow-up by GPs [23, 24].

The proportion of spine patients was higher in PHC than in SHC while it was the opposite for OA and FM patients. Assessment for possible surgery is the most common reason for referral to SHC, but patients are also referred for multidisciplinary examination and/or treatment as well as for applications for disability pension [25]. Most spine patients are diagnosed with unspecific low back pain [26] and the guidelines focus on education, self-management and to stay active [5]. Moreover, surgery is rarely an option [6, 27]. For OA patients however, surgery is the common end-stage with persistent pain, functional loss and advanced radiographic changes [28]. This can explain the difference between the proportions of OA and spine patients treated in SHC. The relatively higher proportion of FM patients treated in SHC compared to PHC is a surprise, given the sparse treatment options for this group in SHC and that surgery is seldom required. However, the high proportion can be explained by the fact that diagnosing FM has been challenging [29]. With a broad variety of symptoms, gradual onset and comorbidities [30], multidisciplinary examination and treatment may be required.

Patients in SHC were older than those in the PHC. Older patients are more prone to comorbidities and to develop chronic pain and this may explain the higher age found in SHC [31]. If these patients did not respond as expected to first-line treatment, the presence of comorbidities may justify referrals to SHC for multidisciplinary examination and/or treatment [5, 16]. The high number of patients in their 40s and 50s could however be a concern. These patients are in their peak earning years and both sick leave and disability pension have high impact on the individual’s income and for the society’s expense. Some of the referrals to SHC for these patients, are probably due to assessment for disability pension, and a need for medical certificate from specialists.

We found that more women than men were seeking treatment for MSD, and the difference increased with increasing age, which is in accordance with results from previous studies [13, 14]. In addition to that women tend to live longer than men, other possible biological and psychological explanations have been proposed. The largest sex difference was among OA and FM patients and can be explained by factors with known differences, like obesity and muscle weakness and menopause [28, 32–34]. Obesity and muscle weakness are important risk factors for developing OA, factors which are more prominent in elderly women (after menopause) than men. The onset of menopause has also been associated with worsening of musculoskeletal symptoms [34]. FM has been considered a female disease and might be diagnosed more easily to women [35]. Moreover, FM can be challenging to diagnose and is often called a diagnosis of exclusion, meaning that the diagnostic process is to rule out other potential causes of symptoms [36]. Hence, more time and health care visits can be required to establish the diagnosis. Since women are more prone than men to visit health care services [37], less men might be diagnosed with FM.

### Patient pathways

Age was similar among patients consulting a GP or PT first. This contrasts with the results from a systematic review on direct access to PT, that patients going directly to PT were younger [38]. However, there are methodological differences. Consulting a PT first in our study, implies a visit to a MT. The MTs role as a primary contact for MSD patients in Norway, with the same rights to refer to specialist care and sick listing patients (up to 12 weeks) as the GP, may be one explanation for this. Hence, we have some selections in our material. We found women to be more prone than men to consult a PT first but have found no other study for comparison. However, it has been reported that women consult PTs more often, overall, than men [39, 40].

In our study, the patients visiting a PT first had fewer consultations by other health care professionals, but the number of PT consultations were much higher, compared to those visiting GPs first. PT consultations are less expensive compared to other health care services, especially in SHC [41], so regardless of the higher number of PT consultations, the costs will probably be lower compared to treatment in SHC. Interestingly, the previous mentioned systematic review found that visiting a PT first reduced the total health care costs and the number of PT consultations [38]. However, methodological differences hamper further comparisons.

In accordance with previous studies [11, 42] most of the MSD patients in our study were low users of health care services and most visited the GP only once. In our analysis of patient pathways, we therefore excluded the low users and included only patients with more than 3 months use. According to Lentz et al. (2019) low users are often younger, have less comorbidities and shorter duration of pain, compared to high users with more severe and longstanding pain and/or disability [10]. All the three diagnosis groups we studied have increased risk of long-term health care utilization compared to several other MSD [26, 43, 44]. Especially OA patients are high users of health care services [6], as we also found in our study. They had the highest number of consultations and were most likely to be treated by several health care professionals compared to the spine and FM patients, though we found no difference in number of GP consultations. Moreover, it is also important to consider that OA patients are most likely to use health care services for a long time. To be able to track the OA patients from the start of symptoms until the end of treatment, a longer follow-up period than two years will be needed.

Referral rates between health care providers often vary between countries due to the different health care systems and referral practice. Reported referral rate will also be affected by study methodology. In our study, we have only information about the treatment rate. Since referral was needed to receive treatment by PTs and in the SHC in the study period, the numbers are most likely comparable. However, referrals can be denied and possibly decrease the treatment rate. The high treatment rate in our study is probably due to the study population, we included only long-term users of health care services in the analyses of patient pathways, and the follow-up time (2 years). This might explain the differences between our results and the lower referral rate found in previous studies from Norway and other Western countries [14, 45]. We found that median time from the first visit in PHC to the first visit to either GP, PT or SHC were more than 6 months and most likely this can be due to long waiting lists. Surprisingly, there was no time difference from the first GP visit to either PT or SHC visits for OA patients. This might indicate that clinical guidelines recommending exercise therapy before further referrals is not followed. Effective treatment pathway also depends on coordination between PHC and SHC. This has been a focus after introducing "The Coordination Reform” 2012 in Norway and health care professionals were encouraged to more interaction.

Our results support that the GPs have a key role for MSD patient in Norway. This is according to clinical guidelines and wanted by Norwegian policymakers: MSD should be managed in the lowest effective care level. The cost for follow-up in primary health care is much lower than getting treatment in specialist health care [41]. After we got the register data for this study, referral to PT was abolished in Norway, and all patients were given direct access. When this is incorporated in the Norwegian health system, we might see an increase of MSD patients visiting a PT first. This might be helpful in reducing the large pressure on the GPs in primary health care.

## Conclusion

One third of the total Norwegian population consulted health care services due to MSD every year in the period 2014–2017. The GP was the most consulted health care professional for MSD patients, while a higher number of consultations were generated from the PT visits, and these trends were consistent during the study period. Among the MSD patients with long-term use of health care services, 50% were treated by more than one health care professional in PHC and/or SHC. OA and FM patients were most and less likely to be referred and treated in SHC, respectively.
